# Correlation between CT Score and KL-6: A Severity Assessing in Juvenile Dermatomyositis Associated Interstitial Lung Disease

**DOI:** 10.1155/2023/5607473

**Published:** 2023-03-27

**Authors:** Chi Wang, Jun Hou, Jianming Lai, Ran Tao, Yang Yang, Wenhan Hao, Xinyu Yuan, Yuchun Yan

**Affiliations:** ^1^Department of Radiology, The Affiliated Children's Hospital, Capital Institute of Pediatrics, Beijing, China; ^2^Department of Rheumatology, The Affiliated Children's Hospital, Capital Institute of Pediatrics, Beijing, China

## Abstract

**Background:**

There is no radiological measurement to estimate the severity of pediatrics juvenile dermatomyositis (JDM) with interstitial lung disease (ILD). We validated the effectiveness of CT scoring assessment in JDM patients with ILD.

**Aim:**

To establish a CT scoring system and calculate CT scores in JDM patients with ILD and to determine its reliability and the correlation with Krebs von den Lungen-6 (KL-6).

**Methods:**

The study totally enrolled 46 JDM-ILD patients and 16 JDM without ILD (non-ILD, NILD) patients. The chest CT images (7.0 ± 3.6 years; 32 male and 30 female) were all analyzed. CT scores of six lung zones were retrospectively calculated, included image pattern score and distribution range score. Image pattern score was defined as follows: increased broncho-vascular bundle (1 point); ground glass opacity (GGO) (2 points); consolidation (3 points); GGO with bronchiectasis (4 points); consolidation with bronchiectasis (5 points); and honeycomb lung (6 points). Distribution range score was defined as no infiltrate (0 point); <30% (1 point); 30%–60% (2 points); and ≥60% (3 points). Two pediatric radiologists reviewed all CT images independently. The ROC curve was established, and the optimal cutoff score for severity discrimination was set.

**Results:**

The agreement between two observers was excellent, and the ICC was 0.930 (95% CI 0.882–0.959, *p* < 0.01). CT score and KL-6 level had a positive linear correlation (*r* = 0.784, *p* < 0.01). However, the correlation between CT scores of different lung zone and KL-6 level was different. The KL-6 cut off level suggested for JDM with ILD was 209.0 U/ml, with 73.9% sensitivity and 87.5% specificity, and the area under curve was (AUC) 0.864 (*p* < 0.01).

**Conclusion:**

The CT scoring system we established, as a semiquantitative method, can effectively evaluate ILD in JDM-PM patients and provide reliable evidence for treatment.

## 1. Introduction

Juvenile dermatomyositis (JDM) is one of the autoimmune diseases in pediatrics, characterized by skin and muscle involvement, which can also affect lung tissue, resulting in interstitial lung disease [1]. The annual incidence of JDM ranges from 2/1,000,000 to 4/1,000,000 [2]. The peak incidence age is from 5 to 10 years, and girls are more susceptible than boys (2–5 : 1) [3–5]. JDM is often associated with interstitial lung disease (ILD), which is difficult to identify early due to its insidious onset. Some of these children can develop severe ILD, which is extremely difficult to treat and has a very high mortality rate. Clinically, serum Krebs von den Lungen-6 (KL-6) and high-resolution CT (HRCT) are often used to evaluate the pulmonary involvement of JDM patients.

KL-6 is an effective biomarker in diagnosing and determining the severity of ILD with connective tissue disease [6]. KL-6 is a high molecular weight mucin-like protein produced by type 2 alveolar lung cells. The increased expression of KL-6 in serum is often associated with damage to alveolar cells. Therefore, the level of KL-6 has a positive correlation with the ILD severity. However, KL-6 has significant limitations in some cases, including its low diagnostic sensitivity. Several factors besides ILD influence KL-6 concentrations, including prolonged smoking, aging [7], renal function [8], and gene (rs4072037) [9].

HRCT is an important supplement for evaluating the degree of ILD. It can offer the possibility of measuring disease severity more accurately and sensitively. However, the ILD lesions of patients were constantly changing. CT could not well evaluate the changes of the patient's condition before and after. Furthermore, different radiologists will give different interpretations for the same signs, particularly in lung imaging. Many scholars have developed multiple systems to standardize the interpretation of CT signs. Evaluating the severity of lung parenchymal involvement based on the common representation in JDM-associated ILD, we reviewed five scoring systems that could quantify the extent of the abnormalities [10–14]. Considering the convenience and feasibility, we designed a 3-grade chest CT severity scoring system to verify the effectiveness of the CT score system and the correlation between KL-6 concentrations. By constructing such a CT scoring system, the lung involvement of patients with JDM can be more accurately evaluated and reliable basis can be provided for follow-up.

## 2. Materials and Methods

### 2.1. Study Population

The Ethics Review Committee of Capital Institute of Pediatric approved this single-center study. This was a retrospective observational study in nature, and the informed consent was exempt. The study was carried out from January 2017 to March 2021 in our institution. Forty six JDM-associated ILD (excluding pulmonary interstitial caused by other diseases) patients underwent 62 HRCT scans. The control group of 16 patients for JDM with non-ILD (NILD) underwent 32 HRCT scans. The cohort of JDM-associated ILD patients in our study had a mean age of (7.0 ± 3.6) years, ranging from 1.5 to 16.0 years, with a nearly equal sex ratio (Table 1). The median follow-up time of CT scan was 6 months. The KL-6 values were measured simultaneously on the day of each CT examination.

### 2.2. CT Protocol

All CT scans were performed on Discovery CT 750 HD (GE Healthcare), from the apex to the base of the lung, with the parameters as followed: pitch 1.375, tube voltages 80 kVp/100 kVp (based on body weights), and rotate time 0.6 s, and the automatic dose modulation technique was used. All the images were reconstructed of 0.625 mm in both thickness and interval space with lung windows (width, 1200 HU; level, −600 HU).

### 2.3. CT Scoring

**Figure 1:**
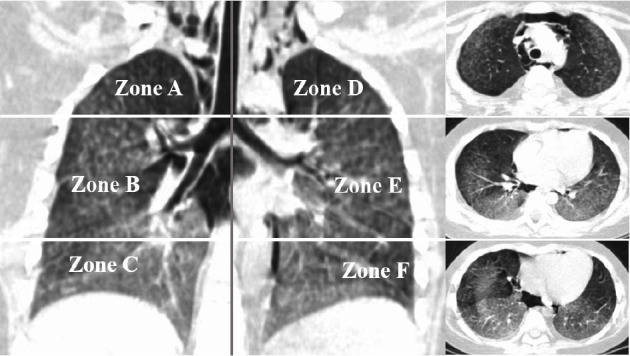
A 9-year-old girl of JDM-ILD. The KL-6 value was 3949 U/ml. The level of pulmonary vein and 1 cm above carina divide the lungs into upper, middle, and lower parts. The right lung was marked from top to bottom as Zone A to C. The left lung was marked from top to bottom as Zone D to F. The score of each zone was equal to image pattern points multiplied by distribution range points. Zone A score = 3 × 2, Zone B score = 3 × 3, Zone C score = 3 × 3, Zone D score = 3 × 2, Zone E score = 3 × 2, and Zone F score = 3 × 2. Total CT score = 42.

The severity and extent of lung involvement were assessed as superimposed in three levels: (1) 1 cm above the carina (upper level), (2) the superior margin of the inferior pulmonary vein (middle level), and (3) inferior pulmonary vein (lower level). The two lungs were divided into six regions. The right lung was marked from top to bottom as Zone A to C. The left lung was marked from top to bottom as Zone D to F (Figure 1).

The score of each zone was equal to image pattern score multiplied by distribution range score. The image pattern score was defined as increased broncho-vascular bundle (1 point); ground glass opacity (GGO) (2 points); consolidation (3 points); GGO with bronchiectasis (4 points); consolidation with bronchiectasis (5 points); and honeycomb lung (6 points). The distribution range score was defined as no infiltrate (0 point); <30% (1 point); 30%–60% (2 points); and ≥60% (3 points) (Figure 1). The total score from Zone A to Zone F was finally summed up. Two pediatric radiologists reviewed all CT images independently. The results were marked as Score 1, Score 2, and Score(mean of Score 1 and Score 2).

### 2.4. Statistics

All statistical analyses were performed with Jeffrey's Amazing Statistics Program (JASP, version 0.14.1). The mean ± standard deviation ($\bar{x}$ ± *s*) is used for all continuous variables that conform to the normal distribution. The anomalous distribution was described by median. The intraclass correlation coefficient (ICC) was used to estimate the agreement of two reviewers. The ROC was used to calculate the sensitivity and specificity. Pearson's correlation analysis was employed to assess the relationship between CT scores and KL-6. A statistical significance was accepted at *p* < 0.05.

## 3. Results

**Figure 2:**
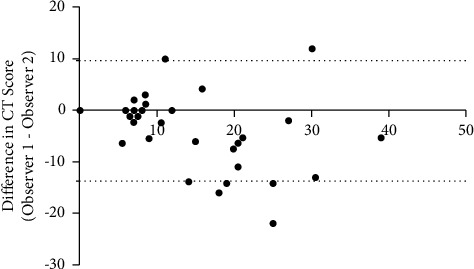
Bland–Altman plots showed better agreement of two independence observers.

The mean CT scores evaluated by radiologist 1 (score 1) and radiologist 2 (score 2) were (11.28 ± 4.47) and (14.07 ± 10.02), respectively, with an excellent agreement evidenced by ICC = 0.930 (95% CI 0.882–0.959, *p* < 0.01). The Bland–Altman test also confirmed that it had good consistency (Figure 2).

**Figure 3:**
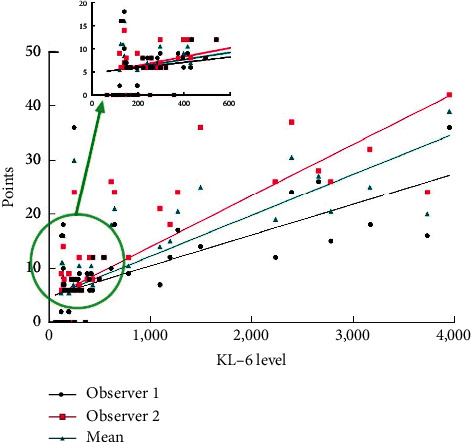
Correlation between different CT scores and KL-6 in JDM children.

**Figure 4:**
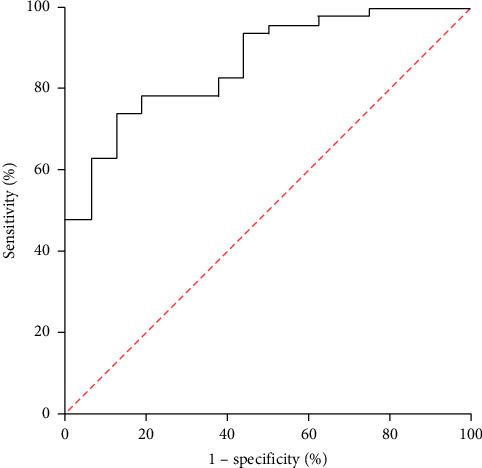
ROC curve of KL-6 level for suggesting ILD in JDM children.

The correlation analysis indicated that the CT score was positively correlated with KL-6, including Score 1 and KL-6 (*r* = 0.716, *p* < 0.01), Score 2 and KL-6 (*r* = 0.820, *p* < 0.01), and Score and KL-6 (*r* = 0.784, *p* < 0.01) (Figure 3). The ROC analysis showed that Youden's index of KL-6 level was 209.0 U/ml, with 73.9% sensitivity and 87.5% area under curve (AUC = 0.864) (95% CI 0.770–0.959) (*p* < 0.01) (Figure 4), respectively.

**Figure 5:**
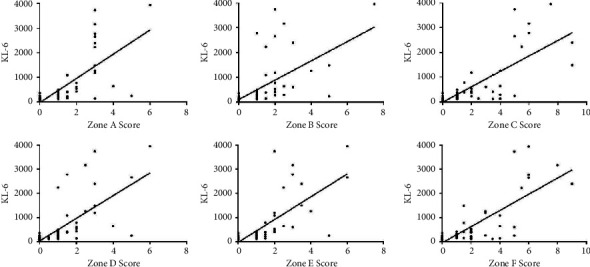
Correlation analysis of CT score and KL-6 from Zone A to F.

**Figure 6:**
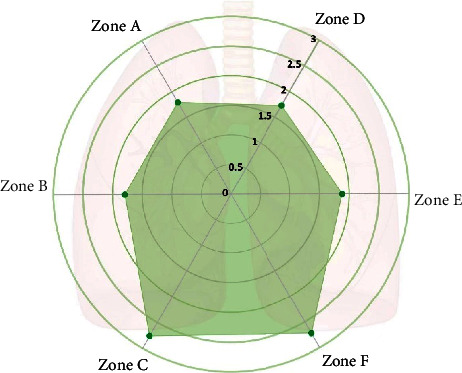
CT score distribution of ILD in JDM-PM patients from Zone A to F.

The correlation between different lung area scores and KL-6 was different, including Zone A (Score = 1.70 ± 1.23, *r* = 0.710, *p* < 0.01), Zone B (Score = 1.79 ± 1.32, *r* = 0.584, *p* < 0.01), Zone C (Score = 2.76 ± 2.21, *r* = 0.762, *p* < 0.01), Zone D (Score = 1.73 ± 1.24, *r* = 0.667, *p* < 0.01), Zone E (Score = 1.88 ± 1.30, *r* = 0.714, *p* < 0.01), and Zone F (Score = 2.72 ± 2.02, *r* = 0.769, *p* < 0.01) (Figures 5 and 6).

## 4. Discussion

HRCT is the gold standard for the diagnosis of ILD, especially for early diagnosis and activity evaluation. Routine chest HRCT findings in DMJ patients are usually warranted due to their relatively high-risk of death. The nonspecific interstitial pneumonia (NSIP) is the main pathological type of JDM-associated ILD patients [15]. NSIP abnormalities on chest CT are mainly located in the lower lung, with diffuse or peripheral distribution in the axial direction. Commonest HRCT signs in NISP are reticular abnormalities, traction bronchiectasis, GGO, and honeycombing [16]. CT scores include an assessment of the extent and severity of lung disease. We selected 3-level chest severity score because it is more intuitive. In our study, there was an excellent agreement among observers which revealed that the 3-level chest severity score was highly reproducible (ICC = 0.930, *p* < 0.01). CT scores were positively correlated with the level of KL-6 (*r* = 0.784, *p* < 0.01). Therefore, CT scores could well reflect the course of ILD in patients. The highest correlation zone we found was the right lower lobe (Zone C) and left lower lobe (Zone F) (*r* = 0.762, *p* < 0.01; *r* = 0.769, *p* < 0.01 retrospectively). In ILD induced by JDM, the lower lobe of the right lung was most seriously involved.

KL-6 is mainly found on the surface of type II alveolar epithelium, especially in the tissue sections of ILD. In ILD patients, the proliferation of type II alveolar epithelium increased the KL-6 expression. KL-6 flows into the bloodstream due to increased permeability following destruction of the alveolar-blood interface. ROC analysis showed the KL-6 level, which can suggest the occurrence of ILD, and the cut off value was higher than 209.0 U/ml (AUC = 0.848, *p* = 0.03). This was lower than the results among connective tissue disease (CTD) patients [6, 14, 17], suggested that JDM may have lower KL-6 levels in the presence of ILD. Therefore, the level of KL-6 is more likely to be affected by other factors, which further reduces its sensitivity. Thus, CT can be an effective supplement to KL-6 in the diagnosis of ILD. The chest CT signs of ILD showed variability, and the half-quantitative method of CT score could effectively evaluate the progression of the disease and provide a reliable basis for treatment.

Inevitably, there are some limitations to this study. The sample size of negative CT findings in this study was small. Moreover, because CT scores are established among experienced radiologists, it needs to be tested in younger radiologists and clinicians. Spirometry is very important for the assessment of lung function [18]; however, young children could not cooperate well, so it was not collected in the study.

## 5. Conclusion

The CT scoring system we established, as a semiquantitative method, can effectively evaluate ILD in JDM-PM patients and provide reliable evidence for treatment.

## Figures and Tables

**Table 1:** Characteristics between ILD group and NILD group (*x* ± *s*).

	ILD (*n* = 46)	NILD (*n* = 16)	Statistics	*p*
Male/female	22/24	10/6	*χ * ^2^ = 1.024	0.312
Age (year)	6.42 ± 3.58	7.13 ± 4.35	*t* = 0.638	0.568
Kl-6 (U/ml)	794.52 ± 150.20	158.06 ± 17.63	*t* = 2.484	0.016
Score 1	11.28 ± 4.47	0	*t* = 6.011	<0.01
Score 2	14.07 ± 10.02	0	*t* = 5.584	<0.01
Mean score	12.67 ± 8.17	0	*t* = 6.169	<0.01

## Data Availability

The data used to support the findings of this study are available from the corresponding author upon request.

## Abbreviations

JDM: Juvenile dermatomyositis
KL-6: Serum Krebs von den Lungen-6
HRCT: High-resolution CT
ILD: Interstitial lung disease
GGO: Ground glass opacity
NSIP: Nonspecific interstitial pneumonia.
